# Seeing red? Colour biases of foraging birds are context dependent

**DOI:** 10.1007/s10071-020-01407-x

**Published:** 2020-07-03

**Authors:** Marianne Teichmann, Rose Thorogood, Liisa Hämäläinen

**Affiliations:** 1 HiLIFE Helsinki Institute of Life Science, University of Helsinki, Helsinki, Finland; 2 Research Programme in Organismal and Evolutionary Biology, Faculty of Biological and Environmental Sciences, University of Helsinki, Helsinki, Finland; 3 Chair of Nature Conservation and Landscape Ecology, University of Freiburg, Freiburg, Germany; 4 Department of Zoology, University of Cambridge, Cambridge, UK; 5 Department of Biological Sciences, Macquarie University, Sydney, NSW Australia; 6 Department of Biological and Environmental Sciences, University of Jyväskylä, Jyväskylä, Finland

**Keywords:** Avoidance learning, Colour preference, Food choice, Great tits, Blue tits, Warning colouration

## Abstract

Colours are commonly used as visual cues when measuring animals’ cognitive abilities. However, animals can have innate biases towards certain colours that depend on ecological and evolutionary contexts, therefore potentially influencing their performance in experiments. For example, when foraging, the colour red can advertise profitable fruits or act as a warning signal about chemically defended prey, and an individual’s propensity to take food of that colour may depend on experience, age or physical condition. Here, we investigate how these contexts influence blue tits’ (*Cyanistes caeruleus*) and great tits’ (*Parus major*) responses to red-coloured almond flakes. We found that juvenile birds preferred red both when it was presented simultaneously with green, and when it was presented with three alternative colours (orange, purple, green). Adult birds, however, only preferred red after a positive experience with the colour, or when it was presented with the three alternative colours. We then tested whether colour influenced avoidance learning about food unpalatability. Despite the prediction that red is a more salient warning signal than green, we found only weak evidence that birds discriminated red unpalatable almonds from a green palatable alternative more quickly than when the colours were reversed. Our results suggest that biases towards red food may depend on birds’ age and previous experience, and this might influence their performance in experiments that use red stimuli. Considering the ecological relevance of colours is, therefore, important when designing experiments that involve colour cues.

**Electronic supplementary material:**

The online version of this article (10.1007/s10071-020-01407-x) contains supplementary material, which is available to authorized users.

## Introduction

Many animal cognition experiments use colour cues to investigate individuals’ performance in different tasks, such as in problem solving, and associative and reversal learning (e.g. Aplin et al. 2015; Morand-Ferron et al. 2015; Shaw et al. 2015; Bebus et al. 2016). However, often these studies do not consider the ecological importance of different colours, which can bias the outcome of the experiment (Rowe and Healy 2014). Red in particular is used as a colour signal in many behavioural studies (e.g. Aplin et al. 2015; Morand-Ferron et al. 2015; Shaw et al. 2015) even though most animals are suspected to have initial biases towards it. Birds, for example, are visual foragers and red colour can provide them information about food profitability. On the one hand, aposematic prey advertise their defences to predators with conspicuous warning signals (Poulton 1890; Mappes et al. 2005; Ruxton et al. 2018), and red is one of the most typical warning colours in many insect orders (Rowe and Halpin 2013) that birds are likely to encounter (Blondel et al. 1991; Naef-Daenzer et al. 2000). On the other hand, in addition to warning avian predators about prey toxicity, red can signal the level of ripeness in fruits and is thought to have evolved to attract avian seed dispersers (Hampe 2001; Schaefer and Schaefer 2006; Albrecht et al. 2012). Birds might, therefore, have context-dependent biases to either avoid or prefer red food, and this might influence their response to red cues in cognitive experiments.

How birds respond to red colour is likely to vary both within and across species, and depend on differences in both evolutionary history (Smith 1975; Schuler and Hesse 1985; Lindström et al. 1999) and individual experience (Schmidt and Schaefer 2004). For example, Schmidt and Schaefer (2004) found that naïve hand-raised juvenile blackcaps (*Sylvia atricapilla*) preferred red artificial fruits to other colours (blue, green, yellow and white), whereas wild-caught adults did not show any preference. This indicates that birds’ innate food colour preferences may change after individuals experience different food types in the wild. Differences in diet and exposure to colours could also explain why avian species are often found to vary in their response to red food. Northern bobwhites (*Colinus virginianus*) and domestic chicks (*Gallus gallus domesticus*), for example, showed an avoidance to red food (Roper 1990; Mastrota and Mench 1995), whereas weka (*Gallirallus australis*) and silvereyes (*Zosterops lateralis*) were found to prefer red (Puckey et al. 1996; Hartley et al. 2000). The food type that individuals encounter might provide another explanation for these inconsistent results. This was demonstrated with domestic chicks and blackcaps that preferred green insects over red ones but did not show colour preferences when presented with green and red fruits (Gamberale-Stille and Tullberg 2001; Gamberale-Stille et al. 2007). This discrimination between food types might be particularly important for generalist foragers whose diet comprises both plant-derived food and animal prey. Failing to recognise aposematic prey might put individuals at risk of consuming toxins, while missing profitable food might lead to a smaller caloric input (Schaefer et al. 2008). In addition to colour, individuals may use several other cues to gather information about food profitability, including odours, tastes, sounds (Rowe and Halpin 2013), and UV reflectance (Siitari et al. 1999). Nevertheless, colour is often found to be the most salient signal for avian species (e.g. Marples et al. 1994).

Potential biases towards red colour may also influence how avian predators learn about prey unpalatability. Indeed, many studies have demonstrated that during avoidance learning birds attend primarily to colour cues (Gamberale-Stille and Guilford 2003; Exnerová et al. 2006; Aronsson and Gamberale-Stille 2008; Halpin et al. 2013). Domestic chicks, for example, pay more attention to the colours of a food item than colour contrast with the background when learning to discriminate palatable and unpalatable food (Gamberale-Stille and Guilford 2003). In addition, both domestic chicks and great tits learn about aposematic insects based on food colour but not pattern (Exnerová et al. 2006; Aronsson and Gamberale-Stille 2008). Likewise, Halpin et al. (2013) demonstrated that European starlings (*Sturnus vulgaris*) learned to associate prey colouration with unpalatability but did not learn to avoid unpalatable prey based on its size. However, studies on the importance of particular colours for discriminative learning have provided inconsistent results. For example, Ham et al. (2006) found that great tits learned to avoid unpalatable artificial prey equally well, regardless of its colour (red, yellow or grey), and Svádová et al. (2009) showed that naïve hand-reared great tits learned to avoid different colour forms of firebugs (*Pyrrhocoris apterus*) at a similar rate. This suggests that the colour which signals unpalatability might be of minor importance for the learning process, as long as unpalatable and palatable cues are distinguishable. However, in contrast to this, Rönkä et al. (2018) found that blue tits (*Cyanistes caeruleus*) learned to avoid the red morph of the aposematic wood tiger moth (*Arctia* *plantaginis*) faster than white or yellow morphs, indicating that red was the most salient warning colour to facilitate avoidance learning. This suggests that colours may have different effects on avoidance learning depending on the bird species and the foraging context.

Here, we test colour preferences and avoidance learning with wild-caught blue tits and great tits. Both species are generalist foragers, with their diet consisting of both arthropods and fruits (Hartley 1953; Betts 1955; Romaya 1970; Albrecht et al. 2012). Our aim was to investigate if birds’ responses towards red food are context dependent and whether red facilitates avoidance learning when food is unpalatable. In the experiments, we offered birds coloured almond flakes to investigate how they respond to a novel food type that is not clearly associated with either profitable fruits or warningly coloured prey. Since birds do not encounter almonds in the wild, their previous experience should not influence their colour choices. Specifically, our aim was to find out:

1. Do birds have an initial bias towards red food, and is this influenced by (i) previous positive experience of red, or (ii) the number and colour of alternative food?
2. Does red facilitate avoidance learning and if so, does this depend on an individual’s initial colour preference?

In the preference tests birds were simultaneously offered several differently coloured almonds on a white tray, ensuring that all colours were equally visible. First, we investigated whether birds preferred red over green almonds, and whether this was influenced by birds’ previous positive experience with red. Since green is both a typical colour of palatable insects and unripe fruits, and red could be interpreted either as a typical insect warning colour or a common colour in fruit displays, birds could be predicted to either prefer or avoid red food. Next, we tested preferences of red versus purple that was assumed to be a more novel colour that birds rarely encounter in the wild. Following this, we added orange (another typical warning colour and a colour of palatable fruits) and presented the four colours simultaneously (red, green, orange and purple). Finally, we investigated whether food colour (red or green) or birds’ initial colour preference influenced avoidance learning when birds were sequentially presented differently coloured palatable and unpalatable food items. For half of the birds red signalled unpalatability and green was palatable; whereas for the other half, the colours were reversed. We predicted that birds would acquire avoidance to red almonds more quickly than to green ones because red is a common warning signal and might be a more salient cue about prey unpalatability.

## Methods

### Birds and housing

We conducted the experiment at the Konnevesi Research Station in Central Finland during November and December 2017. Wild blue tits (n = 38; 24 adults, 14 juveniles) and great tits (n = 39; 12 adults, 27 juveniles) were caught from the feeding site and kept in temporal captivity for the experiments (approximately one week) before being released back at their site of capture. Before release, all birds were ringed for identification. They were also weighed (after capture and before the release) and aged based on their plumage. Birds were housed in individual plywood cages (80 × 65 × 50 cm) with water and food (sunflower seeds, tallow and peanuts) available ad libitum. The cages had automated lights with a daily light period of 12.5 h.

### Food items

Food items were pieces of coloured almond flakes. In the preference tests, we used four different colours: red, orange, green and purple. Almonds were dyed by soaking them for 20 min in a solution of 50 ml water and 0.80 ml (green), 2.50 ml (orange) or 1.25 ml (purple, red) of food dye (Classikool Concentrated Droplet Colours). They were then left air-drying and afterwards cut in small pieces (approximately 3 × 3 mm, 0.1 g). In the avoidance learning test, we used green and red palatable and unpalatable almond pieces. Palatable almonds were prepared using the same protocol as in the preference test. To make the almonds unpalatable, we soaked them for 1 h in a solution of 30 ml of water and 2 g of chloroquine diphosphate, following previously established methods (e.g. Ihalainen et al. 2007). Green or red food dye was added to the solution during the last 20 min before air-drying.

### Preference tests

#### Experimental set-up and training

All experiments were conducted in 50 × 66 × 49 cm sized plywood cages. The cages had a plexiglass front wall which enabled us to observe birds during the trials. Birds were always allowed to habituate to the cage for at least one hour before the experiment was started. Because almond flakes were novel food to the birds, we first presented them with plain (not coloured) almond flake pieces on a white plate. Preference tests were started after birds had finished this training, i.e. when they had consumed all plain almonds.

#### Two colours (red vs. green and red vs. purple)

**Fig. 1:**
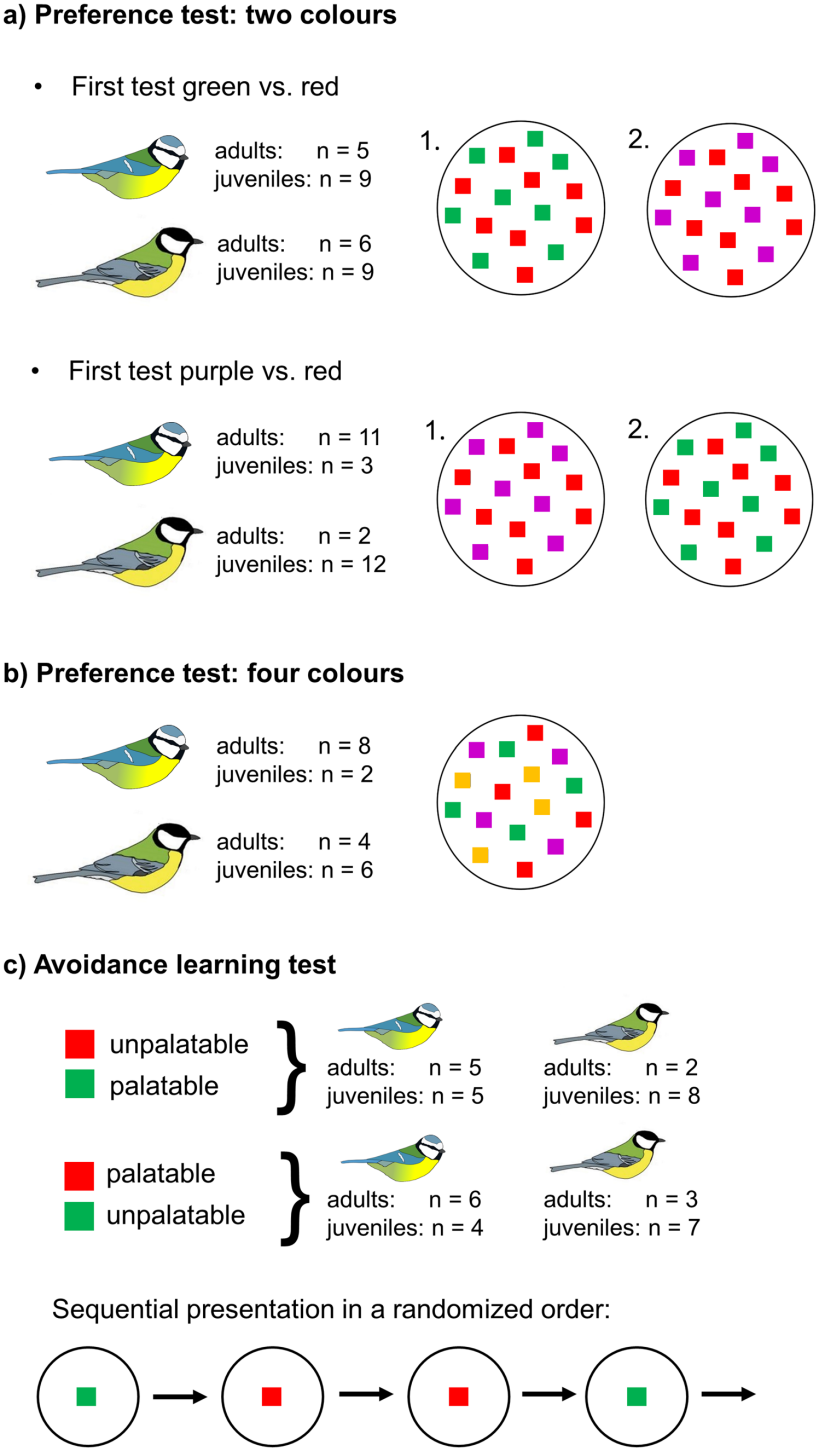
Experimental set-up. In colour preference tests, birds were simultaneously presented with 16 pieces of coloured almond flakes on a white plate. **a** We first tested birds’ preference between red and green, and red and purple, alternating which colour pair was presented first. **b** Another group of birds were then presented with four different colours (red, orange, green, purple). **c** Finally, we tested avoidance learning by offering birds sequentially differently coloured palatable and unpalatable almonds until they rejected the unpalatable colour. The colour that signalled unpalatability (red or green) was randomised among birds

In the first preference test, we investigated if blue tits (*n* = 28; 16 adults and 12 juveniles) and great tits (*n* = 29; 8 adults and 21 juveniles) had initial preferences for (i) red or green, and (ii) red or purple, when the two colours were presented simultaneously. All individuals were tested with both colour pairs and we alternated which pair (red vs. green or red vs. purple) was tested first to investigate how previous experience with red influenced birds’ colour preferences. Therefore, 14 blue tits and 15 great tits were first tested with red and green, and 14 blue tits and 14 great tits first with red and purple (Fig. 1a). We tested the preference by offering birds 8 almond pieces of each colour (i.e. in total 16 pieces). Coloured almond pieces were distributed randomly on a white plate (7 cm diameter) and we recorded the order in which birds chose to eat them. To count as a choice, birds were required to taste the food item instead of just pecking it. If birds did not eat all or any food items within 30 min, we removed the plate for at least 20 min before continuing the trial. Once birds had completed the first pairwise test by eating all 16 food items, they were given a break (at least 30 min) before continuing with the second colour pair.

#### Four colours (red, orange, green, purple)

We next investigated birds’ preferences for red, orange, green and purple almonds when they were all presented at the same time. We used different individuals than in the first preference test, testing 10 blue tits (8 adults and 2 juveniles) and 10 great tits (4 adults and 6 juveniles). We used the same protocol as previously by presenting birds 16 almond pieces (now 4 of each colour; Fig. 1b) on a white plate and recording the order in which birds consumed them. The test was finished when birds had consumed all 16 food items.

### Avoidance learning

In the avoidance learning test, birds were presented with red and green palatable and unpalatable almond pieces, alternating which colour signalled unpalatability. We investigated how fast birds learned to discriminate the colours, i.e. how many unpalatable almond pieces they consumed before rejecting them. We tested 20 blue tits (11 adults, 9 juveniles) and 20 great tits (5 adults, 15 juveniles), all of which had previously participated in the preference tests with two colours (red vs. green and red vs. purple) and, therefore, had experienced palatable red and green almonds. Because birds had consumed more red almonds in the preference test (16 pieces) compared to green almonds (8 pieces), they were offered 8 green almond pieces before the avoidance learning test to ensure equal experience of both colours. In the avoidance learning test, half of the birds (10 blue tits and 10 great tits) were presented with red unpalatable and green palatable almonds, and for the other half, the colours were reversed (Fig. 1c).

In the test, almond pieces were presented sequentially, one piece at a time. Birds were offered 8 pieces (4 palatable and 4 unpalatable) in a semi-randomised order and after that they were given a break (at least 15 min) before continuing with the next 8 food items. To make sure that birds were motivated to forage, the first piece was always a palatable almond, followed by the remaining 7 pieces in a randomised order. Almonds were offered on a white plate and birds were given 3 min to attack (taste and/or eat) the food item. Birds were considered to have learned to discriminate palatable and unpalatable colours when they refused to attack two consecutive unpalatable almonds (within 3 min) but continued to eat palatable almonds that were presented immediately afterwards. The consumption of palatable almonds was important to ensure that birds had learned to discriminate the colours instead of hesitating to attack both colours. If birds did not attack a palatable almond within 3 min, we stopped the trial and waited for them to be more motivated to forage before continuing with the same almond piece. The experiment was continued until birds reached the learning criterion.

### Statistical analyses

We analysed birds’ choices in the preference tests by first calculating a preference score for each colour (Taplin 2007). This was done by ranking the food items from 1 to 16, based on the order that birds consumed them, and then calculating the average rank for each colour. Therefore, small preference scores indicate that birds preferred that colour; whereas, food items with high preference scores were consumed last. We then compared these preference scores in each species using generalised linear models with a preference score as a response variable. Explanatory variables included colour of the almonds (green/orange/red/purple, depending on the preference test), species (blue tit/great tit), and an individual’s age (adult/juvenile). To investigate if colour preferences differed between blue tits and great tits, or between adults and juveniles of each species, we started the model selection with a model that included a three-way interaction term between colour, species and age. When testing birds’ preferences for red vs. green and red vs. purple, each individual was tested consecutively with both colour pairs. Some of the tested individuals therefore had previous experience with red colour, and to test whether this influenced their preferences, these models also included an interaction term between colour and the order of colour pair tests. The terms in the final models were selected based on Akaike’s information criterion corrected for small sample sizes, using MuMIn package (Barton 2019; see Supplementary material for model selections).

We analysed how fast birds learned to discriminate different colours during avoidance learning using a generalised linear model with a poisson error distribution. We compared several models where the number of unpalatable food items attacked before reaching a learning criterion was explained by two-way or three-way interactions between the unpalatable colour (green or red), the species, and an individual’s age, weight and preference score for red (in the red vs. green preference test). This was done to investigate whether the colour that signalled unpalatability influenced avoidance learning, and whether these effects differed between the species or age groups, or instead depended on an individual’s weight or preference for red. The best-fit model was selected based on Akaike’s information criterion corrected for small sample sizes (see Supplementary material for model selection). All analyses were conducted with the software R.3.6.1 (R Core Team 2019).

## Results

### Preference tests

#### Two colours: red vs. green

The preference scores for red and green did not differ between the species (species × colour: estimate = − 1.159 ± 1.013, *t* = − 1.144 *p* = 0.26), but they depended on an individual’s age (age × colour: estimate = − 3.254 ± 0.979, *t* = − 3.324, *p* = 0.001). Furthermore, birds’ preference for red and green was influenced by their previous positive experience of red almonds, i.e. whether they had already participated in the red vs. purple experiment (test order × colour: estimate = − 4.087 ± 0.967, t = − 4.228, *p* < 0.001). Because of this effect of test order, we next ran separate models to investigate (i) birds’ initial biases towards red when they were naïve to both colours (first test red vs. green, *n* = 14 blue tits, 15 great tits) and (ii) preferences of the birds that had already experienced red almonds (first test red vs. purple, *n* = 14 blue tits, 14 great tits).

**Fig. 2:**
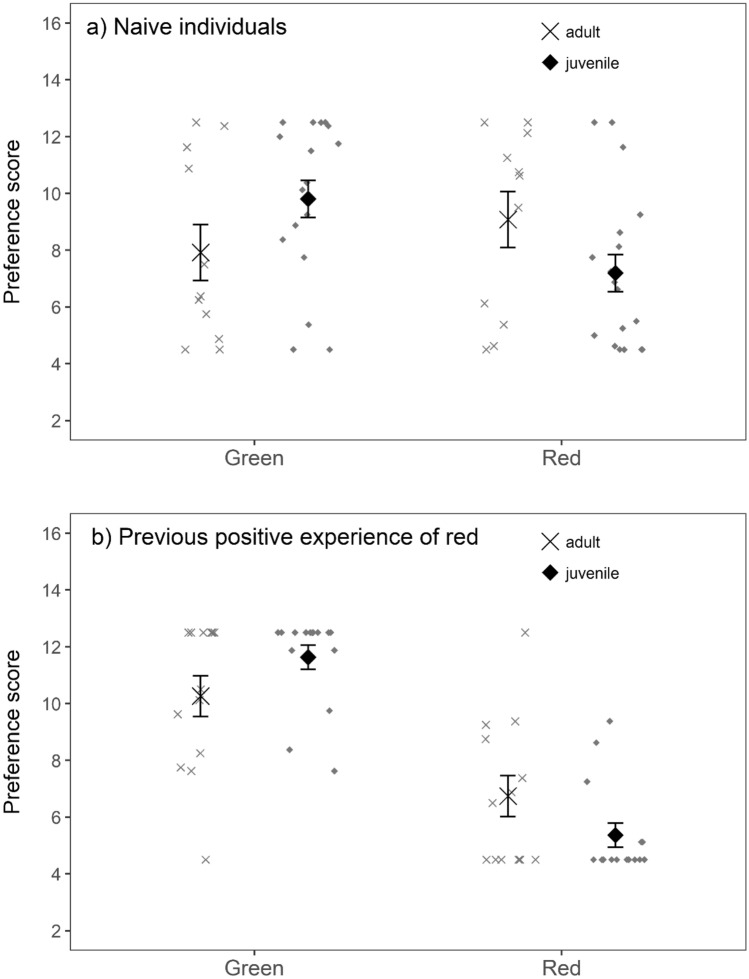
Birds’ preference scores for red and green almonds (**a**) when birds did not have previous experience of coloured almonds (first test red vs. green; *n* = 14 blue tits, 15 great tits), and **b** when birds had previously experienced red almonds (first test red vs. purple; *n* = 14 blue tits, 14 great tits). Smaller preference scores indicate that birds preferred that colour, i.e. consumed it first. The preference scores differed between adults (crosses) and juveniles (black squares). Big symbols show the mean (± s.e.) preference score and smaller symbols present individual variation

When birds were naïve to coloured almonds (when red vs. green was the first test), age had a significant effect on birds’ choices (age × colour: estimate = − 3.770 ± 1.603, *t* = − 2.352, *p* = 0.02). Juveniles preferred red almonds over green (red vs. green: estimate = − 2.611 ± 0.987, *t* = − 2.644, *p* = 0.01), but adults did not show a preference towards either colour (red vs. green: estimate = 1.159 ± 1.263, *t* = 0.918, *p* = 0.36; Fig. 2a). However, if birds already had experience of red almonds (i.e. when red vs. purple was the first test), both juveniles (red vs. green: estimate = − 6.267 ± 0.781, *t* = − 8.025, *p* < 0.001) and adults (red vs. green: estimate = − 3.519 ± 0.839, t = − 4.196, *p* < 0.001) preferred red (Fig. 2b) although this effect was still stronger in juveniles (age × colour: estimate = − 2.747 ± 1.146, *t* = − 2.397, *p* = 0.02).

#### Two colours: red vs purple

Birds preferred red almonds over purple (red vs. purple: estimate = − 2.991 ± 0.419, *t* = − 7.139, *p* < 0.001; see Fig. S1 in Supplementary material), regardless of whether they had previous experience of red almonds (test order × colour: estimate = 1.033 ± 0.858, *t* = 1.205, *p* = 0.23). This did not differ between the species (species × colour: estimate = − 0.018 ± 0.895, *t* = − 0.020, *p* = 0.98), and did not depend on birds’ age (age × colour: estimate = 0.596 ± 0.910, *t* = 0.655, *p* = 0.51), and these interactions were excluded from the final model.

#### Four colours (red, orange, green, purple)

**Fig. 3:**
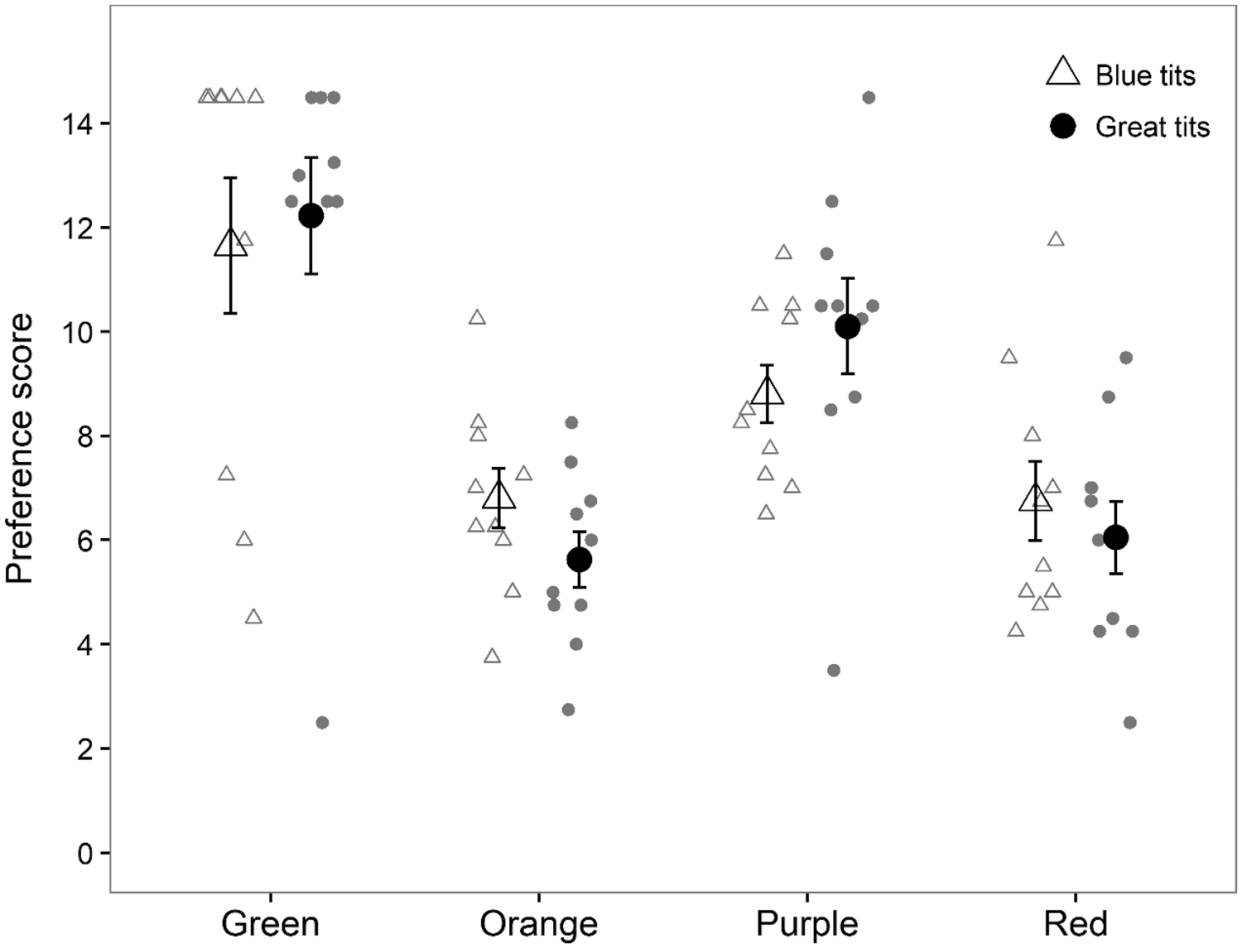
Blue tits’ (open triangles) and great tits’ (black circles) preference scores for differently coloured almonds (*n* = 10 in each species). Smaller preference scores indicate that birds preferred that colour, i.e. consumed it first. Big symbols show the mean (± s.e.) preference score for each species and smaller symbols present individual variation

When presented with four colours, both blue tits and great tits preferred red, orange and purple over green almonds that were consumed last, i.e. the average preference score for green almonds was significantly higher compared to all other colours (orange vs. green: estimate = − 5.725 ± 0.840, *t* = − 6.819, *p* < 0.001; red vs. green: estimate = − 5.538 ± 0.840, *t* = − 6.595, *p* < 0.001; purple vs. green: estimate = − 2.488 ± 0.840, *t* = − 2.963, *p* = 0.004; Fig. 3). There was no difference in preference scores between red and orange almonds (orange vs. red: estimate = 0.188 ± 0.840, *t* = 0.223, *p* = 0.82), and both red and orange were preferred over purple (orange vs. purple: estimate = − 3.238 ± 0.840, *t* = − 3.856, *p* < 0.001; red vs. purple: estimate = − 3.050 ± 0.840, *Z* = − 3.633, *p* < 0.001). The preference scores did not differ between the species or age groups (see Supplementary material for model selection).

### Avoidance learning

**Fig. 4:**
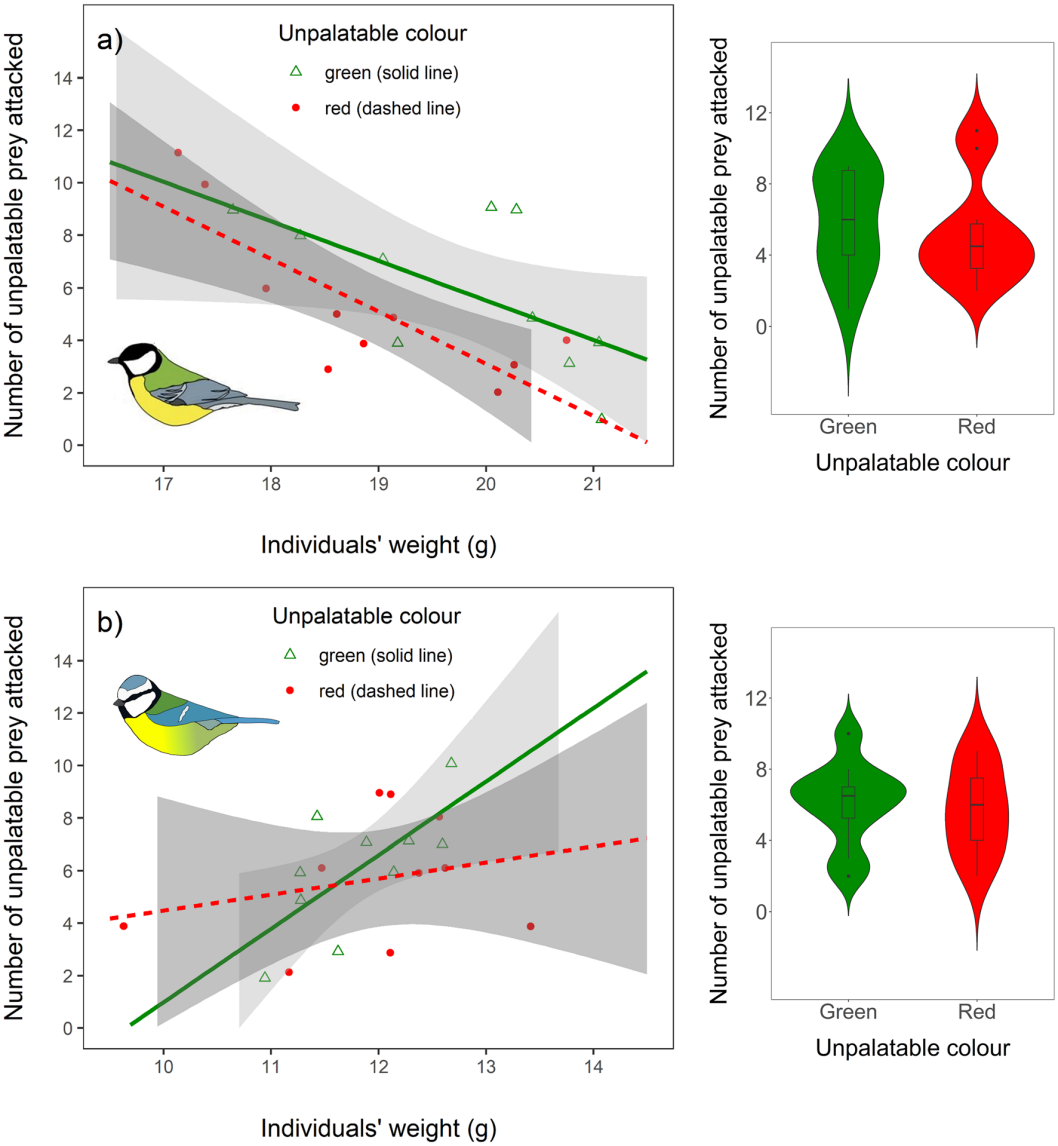
The number of unpalatable food items that **a** great tits (*n* = 20) and **b** blue tits (*n* = 20) attacked during avoidance learning depended on birds’ weight. Half of the individuals were presented with green unpalatable and red palatable almonds (green triangles and solid lines), and for the other half colours were reversed, with red signalling unpalatability (red circles and dashed lines). Shaded areas around the lines indicate 95% confidence intervals for predictions from linear models. Violin plots on the right side of the figure show the overall consumption of unpalatable almonds for each species, according to the colour that signalled unpalatability. Box plots indicate the median and 25th and 75th percentiles, the whiskers show the range of values within 1.5 times the interquartile range and circles are outliers. The violin plot outlines illustrate kernel probability density, i.e. the width of the coloured area represents the proportion of the data located there

The colour used to signal unpalatability (green or red) only had a weak influence on how quickly birds learned to avoid unpalatable almonds, with birds tending to consume fewer unpalatable almonds when they were red (compared to when green signalled unpalatability: estimate = − 0.251 ± 0.140, *Z *= − 1.789, *p* = 0.07; Fig. 4). This effect of unpalatability was not influenced by birds’ initial preference for red (unpalatable colour × preference score for red: estimate = 0.018 ± 0.047, *Z* = 0.379, *p* = 0.70), and it did not differ between the species (unpalatable colour × species: estimate = − 0.181 ± 0.271, *Z* = − 0.668, *p* = 0.50) or age groups (unpalatable colour × age: estimate = − 0.064 ± 0.291, *Z* = − 0.221, *p* = 0.83), so these interaction terms were removed from the final model. Interestingly, we found that the number of unpalatable almonds attacked depended on an individual’s weight, but this effect varied between the species (species × weight: estimate = − 0.538 ± 0.157, *Z* = − 3.426, *p* < 0.001). Irrespective of almond colour (unpalatable colour × species × weight: estimate = 0.183 ± 0.323, *Z* = 0.567, *p* = 0.57; unpalatable colour × weight: estimate = − 0.027 ± 0.036, *Z* = − 0.771, *p* = 0.44), great tits attacked more unpalatable almonds when their body weight was low (effect of weight: estimate = − 0.298 ± 0.080, *Z* =  −3.727, *p* < 0.001; Fig. 4a); whereas, the opposite was true in blue tits, with the tendency of heavier birds to attack more unpalatable food items (effect of weight: estimate = 0.240 ± 0.130,  *Z* = 1.840, *p* = 0.07; Fig. 4b). An individual’s age did not influence the number of unpalatable almonds it attacked (estimate = 0.094 ± 0.144, *Z* = 0.651, *p* = 0.52).

## Discussion

Colour cues are commonly used in animal cognition studies (Aplin et al. 2015; Morand-Ferron et al. 2015; Shaw et al. 2015; Bebus et al. 2016), but potential biases towards different colours remain poorly understood. Here we investigated context-dependent food colour biases in two generalist avian species. We found that both blue tits and great tits preferred red colour in a positive context, but this also depended on an individual’s age and previous experience. Juveniles of both species preferred red over green almonds regardless of their prior experience, whereas adults chose red before green only after having positive experience of it. Both adults and juveniles, however, preferred red and orange over green and purple when the four colours were presented simultaneously, and they also preferred red over purple in the pairwise test. We found only weak evidence that colour influenced avoidance learning about unpalatable food, with a non-significant trend for birds to consume fewer unpalatable almonds when unpalatability was associated with red colour instead of green. In great tits, also physiological condition affected an individual’s tendency to attack unpalatable prey, with lighter individuals consuming more unpalatable almonds compared to heavier birds. Our results support the idea that red can act as a positive stimulus for omnivorous birds but this may depend on an individual’s age and previous experience with the colours.

During preference tests, birds predominantly preferred red almonds over alternative colours, which suggests that they perceived almonds more as a fruit-like food than warningly coloured prey. This preference has also been demonstrated in naïve juvenile blackcaps and redwings (*Turdus iliacus*) when choosing from differently coloured fruits (Honkavaara et al. 2004; Schmidt and Schaefer 2004) and it is further underpinned by studies with several other bird species (e.g. Willson et al. 1990; Willson 1994; Puckey et al. 1996; Hartley et al. 2000). In contrast, Ham et al. (2006) found that great tits chose red least often compared to orange, yellow and grey food. This opposite result could be explained by a different presentation of food colours. Compared to our study where almonds were dyed, Ham et al. (2006) covered peanuts with coloured paper, which could have influenced how birds perceived them.

Another explanation for the differences we observed with previous studies was that the birds’ preference for red may vary across seasons and years depending on the abundance of red food types in the environment (Hartley 1953; Betts 1955). Our experiment was conducted during a mast season of European rowan (*Sorbus aucuparia*) (e.g. Fox et al. 2009) that was fruiting abundantly on the premises of the research station. Both blue tits and great tits feed on its red pulp (Albrecht et al. 2012). The birds were, therefore, likely to have gained recent positive experience of red fruits in the wild, potentially influencing their colour preferences in the experiments. When presented with three alternative colours, birds did not differentiate between red and orange almonds, which indicates that they perceived both colours as equally profitable. Contrary to the assumption that darker fruits have a higher nutrient and antioxidant content (Willson 1994; Schaefer et al. 2003, 2008), birds also preferred red over purple. It is possible that purple is a more novel colour that blue tits and great tits rarely encounter in the wild, which could have explained their hesitation to consume it (Marples and Kelly 1999).

Interestingly, the preference for red palatable almonds was less pronounced in adult birds compared to juveniles. When presented with red and green almonds, adults favoured red only after having previous experience of its palatability in contrast to juveniles that always preferred red over green. This is similar to a previous study which found that juvenile blackcaps preferred red food, while adults did not show any colour preferences (Schmidt and Schaefer 2004). However, blackcap juveniles were hand-raised, and, therefore, differed from wild-caught adults in their previous exposure to different foods; whereas, in our experiments, both juveniles and adults were captured from the wild. Nevertheless, food availability varies across seasons and adult birds were likely to have gained more experience of different food types with varying palatability, which could have increased their hesitation to consume red food. Following this, it is also possible that recent positive experience with the fruits of European rowan at the study site had a greater influence on the preferences of less-experienced juveniles. Another possible explanation for the observed age differences is that there is actually an innate preference for red, which might be overcome later in life when individuals experience different food types that vary in palatability (Schmidt and Schaefer 2004).

Although red is a typical warning colour in aposematic insects (Rowe and Halpin 2013), evidence that it influences avoidance learning is mixed. For example, previous studies with great tits have found no effect of prey colour on the speed at which the birds learned to avoid unpalatable prey (Ham et al. 2006; Svádová et al. 2009), while a study with blue tits found the opposite (Rönkä et al. 2018). Our results do not help to resolve this conundrum as we found only marginal evidence that red colouration might influence avoidance learning when combined with distastefulness. Prey types in previous studies have varied from completely artificial prey similar to our study (coloured paper prey; Ham et al. 2006) to biologically informed models (‘moth wings’ resembling target species in size, shape and spectral reflectance; Rönkä et al. 2018) and real insects (firebugs; Svádová et al. 2009), which could explain why these studies have not found consistent effects of colour on avoidance learning. Indeed, it is possible that the specific colour that signals unpalatability does not strongly influence prey discrimination but that its salience depends on multiple signal components (Rowe and Halpin 2013). Interestingly, we found that great tits consumed more unpalatable almonds when they were lighter, irrespective of which colour indicated unpalatability. This supports the idea that birds are more willing to consume chemically defended prey when they are in a poorer physiological condition (Barnett et al. 2007, 2012; Skelhorn et al. 2016; Hämäläinen et al. 2020a). In contrast, we found the opposite effect in blue tits, with heavier individuals tending to attack more unpalatable food items than lighter birds. This suggests that the costs of consuming chemically defended prey may differ between the two species (Hämäläinen et al. 2020b), and the smaller body mass of blue tits could make them more susceptible to prey toxins compared to larger great tits that might be able to cope with higher toxin loads.

Our study shows that food colour preferences in wild omnivorous birds vary due to an individual’s age and previous experience, and to a lesser extent, the palatability of the food. This could potentially influence the outcome of cognitive studies that often use colour cues to investigate discriminative learning (e.g. Morand-Ferron et al. 2015; Shaw et al. 2015; Bebus et al. 2016), or studies designed to test avoidance learning with coloured stimuli where the age or prior experience of the test subjects is unknown (e.g. studies with wild-caught birds, Ham et al. 2006; Halpin et al. 2013; Rönkä et al. 2018). It could also explain why previous studies on birds’ food colour preferences have found variable results (Willson et al. 1990; Willson 1994; Mastrota and Mench 1995; Puckey et al. 1996; Hartley et al. 2000; Gamberale-Stille and Tullberg 2001; Ham et al. 2006; Gamberale-Stille et al. 2007; Rönkä et al. 2018). Future studies should, therefore, take into account several potential signalling functions of colours and consider how this may influence an animal’s behaviour in cognitive tasks.

## Electronic supplementary material

Below is the link to the electronic supplementary material.

Supplementary material 1 (XLSX 26 kb)

## Data Availability

All data are available in the Supplementary material.
